# Slippery Stents: A Case Report and Review of the Literature Describing Patients with May-Thurner Syndrome That Experienced Stent Migration

**DOI:** 10.1155/2019/7606727

**Published:** 2019-03-05

**Authors:** Ramy Mando, Priscilla Sigua-Arce, Lisa Spencer, Alexandra Halalau

**Affiliations:** ^1^Department of Internal Medicine, Beaumont Health System, Royal Oak, MI, USA; ^2^Oakland University William Beaumont School of Medicine, Rochester Hills, MI, USA

## Abstract

Endovascular stent placement is an effective treatment for relieving chronic venous obstruction in patients with May-Thurner Syndrome (MTS) with or without the presence of thrombotic lesions. Stent migration is a rare but potentially life-threatening complication of endovascular stenting. Herein, we describe a case of stent migration from the left common iliac vein into the right heart, requiring open-heart surgery. We also completed a literature review of MTS patients with stent migration in hopes of raising awareness of this rare and life-threatening complication.

## 1. Introduction

May-Thurner Syndrome was first described in 1908 and was not completely understood until the mid-1900s [1, 2]. The majority of cases described in the literature identify MTS as compression of the left common iliac vein by the right common iliac artery against the lumber vertebrae; however, variants do exist; for example, compression of the inferior vena cava by the right iliac artery has been described and also termed NIVL or nonthrombotic iliac vein lesions [3–5]. Therefore, MTS has been described as an obstruction of venous outflow anatomically secondary to arterial compression in the iliocaval region. 

While many patients with MTS present with left lower extremity deep vein thrombosis (DVT), symptoms can include swelling, pain, claudication, ulcerations, varicose veins, and pelvic congestion syndrome [6, 7]. The use of stents in MTS has been found to be safe and efficacious for patients with postthrombotic syndrome, as well as those presenting with edema [8]. Stent migration is a rare, but potentially fatal complication of the procedure. Few cases of stent migration from iliac veins to the heart have been reported [9–11]. Here we present a case of stent migration to the heart following placement in the left iliac vein for treatment of May-Thurner syndrome.

## 2. Case Description

**Figure 1:**
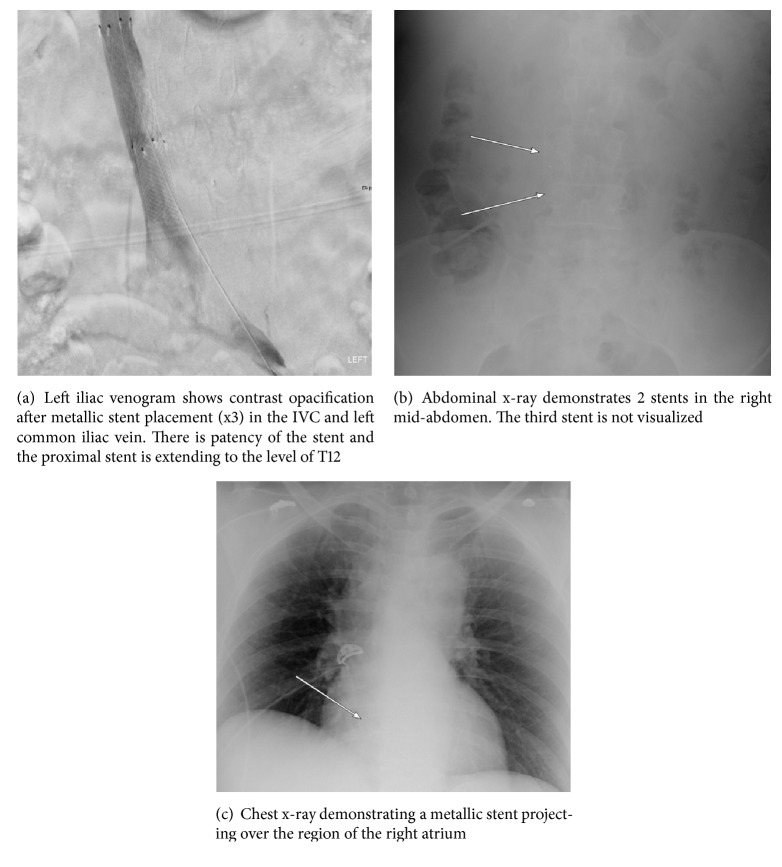


A 62-year-old female presented for evaluation of recurrent left lower extremity swelling. Her medical history was notable for prior deep vein thrombus in the right distal lower extremity while on hormone replacement therapy (HRT). She denied the active use of HRT and tobacco use during this admission. Venous Doppler ultrasound completed in the emergency room revealed extensive thrombosis of the left lower extremity extending superiorly towards the left common iliac vein. Further imaging with ultrasound revealed compression of the left iliac vein by the right iliac artery as well as a significantly elevated reflux time of the left great saphenous vein (14.2 seconds) suggestive of MTS. The patient was taken to the operating suite and during the procedure the common iliac vein appeared normal distally, but more proximally the vein was narrowed significantly to a diameter of less than 2 mm. Prior to entering the inferior vena cava, the common iliac vein normalized. Using intravenous ultrasound, measurements were taken and a 14 x 60 mm Luminexx stent was deployed at the area of stenosis. The stent was noted to have migrated upward into the inferior vena cava and a buttressing of this stent with a 16 x 40 mm Wallstent was placed to ensure adequate apposition. Unfortunately, this caused further migration upward into the IVC and a 14 mm Atlas balloon was used to help secure the migrated IVC stent. The area of stenosis was no longer stented given this migration. Therefore, stenting of the left common iliac vein stenosis was ultimately achieved with a 14 x 80 mm Luminexx stent (Figure 1(a)). The patient was started on warfarin with heparin bridging postoperatively. Early ambulation and the routine use of elastic stockings were encouraged following the procedure. The following day the patient complained of severe abdominal pain and an abdominal x-ray revealed only two stents located in the abdomen (Figure 1(b)). A chest x-ray was obtained and revealed the initial 14 x 60 mm Luminexx stent projecting over the right atrium (Figure 1(c)). She underwent open-heart surgery for stent retrieval and had a postoperative course complicated by atrial fibrillation and recurrent left sided lower extremity DVT managed with catheter directed thrombolysis. Hypercoagulable work-up revealed homozygosity of the Factor V Leiden gene mutation. One week after discharge, she developed hypotension and lightheadedness. She presented to the emergency department and was found to have pericardial tamponade requiring blood transfusion, pericardiocentesis, and pericardial window. Anticoagulation treatment was stopped during hospital stay and not resumed upon discharge.

Three weeks later, she had a syncopal episode secondary to a massive pulmonary embolus (PE). Imaging also revealed residual DVT in bilateral lower extremities. She underwent thrombolysis with tissue plasminogen activator and subsequently developed a thoracic hematoma. Given the residual clot burden in the bilateral lower extremity, she underwent IVC filter placement and mechanical thrombectomy. No additional stents were placed. Throughout the hospitalization the patient required multiple blood products after developing a hematoma related to recent thoracic surgery. The patient was eventually stabilized and given the Factor V Leiden mutation and life-threatening PE, she was started on rivaroxaban indefinitely. Since these events, she has been followed closely as an outpatient with no known hospitalizations related to bleeding or thrombosis. At 5-year follow-up, the patient reports that she is doing well. She is not experiencing any complications related to rivaroxaban. She does have residual postthrombotic syndrome (CEAP class 3, Villalta Score 8) well managed with daily compression stockings.

## 3. Literature Review

A systematic review of PubMed and MEDLINE was performed without any search restrictions. We used the keywords “stent”, “migration”, “iliocaval obstruction”, “iliac vein compression”, “iliac stent”, and “May Thurner”. We screened the reference sections of the eligible results for any potential missed cases of relevance to ensure the inclusion of all reported cases of stent migration in patients with MTS treated with endovascular stenting. Our search yielded six manuscripts. All manuscripts (four out of six results) describing cases of stent migration were included in our review and are summarized in Table 1.

The literature review is notable for varying frequencies of stent migration ranging from 1.4 to 6.25% [12–14]. Three patients (out of 11 patients total) were identified in the literature review as having stent migration to the heart [9, 11, 12]. One patient was managed initially with open-heart surgery for stent retrieval. Initial snare recovery of the stent was attempted in two patients; one resulted in stent fracture and subsequent open-heart surgery [9] and one patient had an uncomplicated retrieval. All patients who underwent open-heart surgery (3/11) experienced significant postoperative complications including stroke, atrial fibrillation, tamponade, chord rupture, and leaflet damage requiring valve replacement in some instances [9, 11, 12]. There was no associated mortality with any of these procedures.

## 4. Discussion

Our case describes a patient with MTS that presented with an acute deep vein thrombosis that was treated with endovascular stenting and, following stent placement, experienced stent migration to the heart and subsequent open-heart surgery for stent retrieval. Her postoperative course was complicated primarily by cardiac tamponade, massive pulmonary emboli, and recurrent DVTs. Our literature review highlights the significant morbidity associated with open-heart surgery and strongly favors stent retrieval utilizing snares. Overall, this case highlights the necessity for establishing a standardized safe management algorithm for patients with MTS. Furthermore, it highlights the importance of assessing risk associated with each treatment option and the clinicians' responsibility to be prepared for complications related to all procedures to help ensure patient safety.

There is currently no expert consensus or guidelines available to the medical community to help direct the management of this condition. Therapeutic options for MTS complicated by acute DVT or postthrombotic syndrome described in the literature include endovenous treatment with endovascular stenting, catheter-directed thrombolysis, pharmacologic thrombolysis, and surgical exploration of the lesion with thrombectomy and decompression of the vein. Overall, several case reports and retrospective reviews have exhibited that endovascular, minimally invasive techniques are relatively safe and provide good symptomatic improvement [16–18]. When compared to oral anticoagulation alone, catheter-directed thrombolysis has been shown to be superior with regard to resolution of the thrombus and rates of postthrombotic syndrome [18]. With regard to angioplasty, endovascular stenting appears to have a role in patients with MTS, irrespective of the presence of acute thrombus [19–22].

Symptoms related to complicated MTS include lower extremity swelling, venous claudication, chronic venous insufficiency, pulmonary embolism, and pelvic congestion syndrome. We believe MTS to be underdiagnosed clinically. We feel that part of the reason this condition is underdiagnosed clinically may be due to lack of physician awareness of this pathology. It is important to recognize these patients as treatment options are available to them which may relieve many of their symptoms and prevent symptom recurrence. Although management options appear straightforward, it is also important for clinicians to recognize potential complications related to treatment, particularly stent migration. Other complications related to stent placement include thrombosis, stenosis, and fracture. Stent migration, however, may lead to the greatest morbidity in these patients, as seen in our case. We also note that proximal migration of the stent within the vessel may occur and appears to be well managed with simultaneous placement of another stent which overlaps the distal portion of the proximally migrated stent [13, 14]. Our case of stent migration to the heart is the fourth case in the literature. Overall of these four cases, two patients underwent heart surgery for stent recovery, one stent retrieval was successfully completed with snare and one failed snare retrieval and underwent heart surgery [9, 11]. Given the significant complications associated with surgical retrieval (atrial fibrillation, stroke, tamponade, leaflet destruction, and chord rupture), we feel that initial recovery attempts should be with snares and surgical recovery should be reserved for those who fail this therapy. One suggestion, which may help avoid this significant complication, may be the use of self-expanding bare metal stents as these typically provide better apposition against the vessel wall given their superior flexibility [12].

## 5. Conclusion

Stent migration may occur in as many as 6.25% of cases and typically leads to significant morbidity if retrieval requires open-heart surgery. We recommend the use of a snare for stent retrieval as this appears to be associated with far fewer complications when compared to open heart surgery based on the limited reports available.

## Figures and Tables

**Table 1:** Characteristics and outcomes of patients with MTS and stent migration.

Article	Description	Interventions and Complications	Outcome
(1) Elmahady S, et.al. [9]	65-year-old patient, with MTS stenting six months prior, presented with signs and symptoms of acute heart failure; transthoracic echocardiogram showed foreign body within right ventricle; transesophageal echocardiogram showed long stent straddled the tricuspid valve.	Percutaneous endovascular approach with snare was tried, it fractured the stent, leaving two segments. Surgical extraction was performed; as chords and leaflets were ruptured, valve replacement was done. Developed hemopericardium secondary to anticoagulation and small thromboembolic cerebellar stroke from atrial fibrillation.	Good prognosis at 8-month follow-up.

(2) Huang C, et al. [12]	68 patients with MTS stenting; 65 underwent stent implantation and 3 underwent simple balloon angioplasty. 75 stents were used among 65 patients; in 1 patient (1.5%), migration of the stent to the right ventricle was reported.	For the patient who had a stent migration to the right ventricle, a snare was successfully used.	Developed iliac vein occlusion within a month. The patient declined intervention.

(3) Mullens W. et. al [11]	55-year-old patient, who had two stents placed a year before in left iliac vein, presented with progressive dyspnea and fatigue. Transesophageal echocardiogram showed severe tricuspid regurgitation and two stents in the right ventricle.	Surgical removal of the stents was performed; as leaflets and chords were severely damaged, a tricuspid valvuloplasty was performed. No postoperative complications were reported.	Not reported.

(4) Ye K, et al. [13]	205 patients with MTS underwent stent implantation. 227 stents were used. 17 patients required two stents for treatment, 3 (1.4%) of these patients required two stents due to migration of the first stent to the proximal segment of the compressed iliac vein.	For the 3 patients (1.4%) who had stent migration to the proximal segment of the compressed vessel, a second stent, that overlapped with the distal segment of the first stent, was used. No complications were reported.	Specific outcome for these 3 patients was not reported.

(5) Liu Z, et. al [14]	48 patients with MTS underwent stent implantation. 49 stents were used. 3 (6.25%) patients required two stents for treatment as the initial stents migrated proximally.	For the 3 patients (6.25%) who had stents migration to the proximal segment of the compressed vessel, a second kissing stent was placed. No complications were reported.	Specific outcome for these 3 patients was not reported. No stent fractures or migrations were reported at 1-year follow-up.

(6) Hartung O, et. al. [15]	89 patients with non-malignant obstructive iliocaval lesions, 52 of which were classified as MTS, underwent stent implantation. 2 (2.24%) patients had stent migration	One patient had a stent migrate into the retrohepatic IVC: it was pulled down into the infrarenal IVC, where it adopted a transversal position; another stent was deployed in the iliocaval junction. The second patient's stent was pulled back from the right atrium down to the left CFV with two Amplatz Goose Neck Snare kits (ev3, Inc, Plymouth, Minn), where it was retrieved surgically.	The patient was asymptomatic 33 months later, and the stent did not migrate. The second patient had another stent accurately deployed 3 months later.
